# A Clinical and Confocal Microscopic Comparison of Transepithelial PRK and LASEK for Myopia

**DOI:** 10.1155/2014/784185

**Published:** 2014-07-10

**Authors:** Safak Korkmaz, Kamil Bilgihan, Sabahattin Sul, Ahmet Hondur

**Affiliations:** ^1^Department of Ophthalmology, Düzce State Hospital, Düzce, Turkey; ^2^Department of Ophthalmology, Gazi University Medical School, Ankara, Turkey; ^3^Department of Ophthalmology, Yatağan State Hospital, 48500 Muğla, Turkey

## Abstract

*Purpose*. To compare the clinical and confocal microscopic results of transepithelial PRK versus LASEK for correction of myopia. *Materials and Methods*. Twelve patients with myopia received transepithelial PRK in one eye and LASEK in the other. In transepithelial PRK-treated eyes, the corneal epithelium was removed with 40 microns of excimer laser ablation and in LASEK-treated eyes with 25-second application of 18% ethanol. Time to epithelial healing, ocular discomfort, uncorrected and best corrected visual acuities, manifest refraction, haze, greyscale value, and keratocyte apoptosis in confocal microscopy were recorded. *Results*. The mean time to epithelial healing was significantly longer after LASEK (4.00 ± 0.43 versus 3.17 ± 0.6 days). On day 1, ocular discomfort was significantly higher after transepithelial PRK. The grade of haze, keratocyte apoptosis, and greyscale value in confocal microscopy were significantly higher in transepithelial PRK-treated eyes at 1 month. All transepithelial PRK- and LASEK-treated eyes achieved 20/25 or better UCVA and were within ±1.00 D of emmetropia at final visits. *Conclusions*. Both transepithelial PRK and LASEK offer effective correction of myopia at 1 year. However, LASEK appeared to induce less discomfort and less intense wound healing in the early postoperative period.

## 1. Introduction 

Laser-assisted subepithelial keratomileusis (LASEK) was introduced by Massimo Camellin (M. Cimberle, “LASEK May Offer the Advantages of Both LASIK and PRK,” Ocular Surgery News, International Edition, March 1999, page 28) as a technique which would eliminate the disadvantages of photorefractive keratectomy (PRK) and laser in situ keratomileusis (LASIK). The theoretical advantages ascribed to LASEK were less postoperative pain, faster visual recovery, and less haze than PRK. However, clinical studies comparing PRK and LASEK have yielded controversial results in terms of postoperative pain, speed of visual recovery, and wound healing [1–5]. Transepithelial PRK has been also associated with diminished wound healing response, hence, less refractive regression and haze compared to other techniques of epithelial removal in PRK [6, 7].

The aim of the present study is to evaluate and compare clinical and confocal microscopic findings after transepithelial PRK and LASEK for correction of myopia as both techniques have beneficial effects on corneal wound healing.

## 2. Materials and Methods

Twelve consecutive patients (3 men, 9 women) with less than 0.5 diopter (D) differences in myopic spherical equivalent (SE) refraction and astigmatism between their eyes were included in this study. Data of the study were retrospectively collected. The age of patients ranged between 19 and 32 years.

The refractive error was treated with transepithelial PRK in one eye and LASEK in the other eye of each patient. Odd numbered patients received transepithelial PRK in the right and LASEK in the left eye and vice versa in the even numbered patients. Written informed consent was obtained from all patients. The tenets of Declaration of Helsinki were followed throughout the study.

Inclusion criteria were at least 18 years of age, stable refraction of at least 2 year, and normal corneal topography. Daily-wear soft contact lenses were removed at least 2 weeks before the preoperative examination. Preoperative evaluation included medical history and complete ophthalmologic examination (uncorrected visual acuity (UCVA), best spectacle-corrected visual acuity (BSCVA), manifest and cycloplegic refractions, anterior segment examination, applanation tonometry, ophthalmoscopy, corneal topography, pachymetry, Schirmer testing, and confocal microscopy). Patients with unstable refraction, dry eye, blepharitis, corneal disease, glaucoma, collagen vascular disease, diabetes, and topographical evidence of keratoconus were excluded.

All laser procedures were performed by the same surgeon. All patients were treated bilaterally, with both eyes treated at the same surgical session. All procedures were performed under sterile conditions in an operating room environment. Topical proparacaine 0.5% was used to anesthetize the eyes. A drape and a lid speculum were inserted following the treatment of eyelids with 10% povidone-iodine.

In transepithelial PRK-treated eyes, initially the epithelium was ablated using the phototherapeutic keratectomy (PTK) mode with laser ablation set to 8.0 mm diameter and 40 *µ*m depth. This step was performed with all lights in the operation room turned off to observe the disappearance of blue fluorescent light of the epithelium. As soon as the blue fluorescence of the epithelium disappeared, the laser was immediately switched to the refractive correction program and stromal ablation was performed without delay.

In LASEK-treated eyes, the epithelium was incised with an 8 mm trephine placed centrally, and 18% alcohol was applied for 25 seconds. The epithelium was detached and gathered at 12 o'clock.

Laser ablation was performed with the ESIRIS excimer laser (SCHWIND, Kleinostheim, Germany). Spherical and cylindrical ablations were performed according to manifest refraction without any reduction using the SCHWIND ORK-CAM aspheric ablation profile. The ablation diameter was 6.5 mm with a 0.75 mm transition zone in all eyes. Following the ablation, the cornea was irrigated with chilled balanced salt solution, and in LASEK-treated eyes the epithelium was rolled to its original position and dried in place for 2 minutes. A cooled soft contact lens (Focus Night & Day; Ciba Vision, Duluth, Ga) was placed over the cornea with sterile forceps, and a drop of tobramycin 0.3% and dexamethasone 0.1% were instilled. The eyelid speculum and drape were removed.

Patients were examined daily until epithelial closure and at 1, 3, 6, and 12 months. Postoperative medication until epithelial closure consisted of topical tobramycin and dexamethasone five times daily. Diclofenac 50 mg was prescribed to all patients, and they were advised to take it orally once or twice per day if required.

The contact lenses were removed after epithelial closure. Topical tobramycin was discontinued following epithelial closure. Dexamethasone was administered four times daily for 1 month followed by fluorometholone 0.1% four times daily for another 1 or 2 months depending on refraction. All medications were discontinued after 3 months.

All patients were given a questionnaire on the day of surgery and asked to rate and compare their pain levels in each eye on days 1 and 2. The pain scale was defined as follows: level 1: no pain; level 2: minimal pain; level 3: moderate pain; level 4: severe pain; and level 5: unbearable pain. Pain level was not rated after third day as the epithelium had healed in some PRK treated eyes on day 3.

Postoperative haze was graded as follows: +0.5: barely visible corneal opacity; +1: reticular subepithelial opacities not affecting visual acuity; +2: punctuate or coalesced subepithelial opacities affecting visual acuity; +3: confluent subepithelial opacities affecting visual acuity and partially obscuring iris detail; and +4: dense opacities completely obscuring iris detail.

Greyscale value and keratocyte count of the anterior stroma immediately beneath the epithelium was evaluated with the Confoscan 3 confocal microscope (NIDEK Technologies, Padova, Italy) at 1, 3, and 6 months. Coronal section of each image was approximately 340 *µ*m vertically and 255 *µ*m horizontally. Each image was separated from the adjacent image by an average of 6 *µ*. The lateral resolution was 1 *µ* and depth of field was 10 *µ* for each image. To determine the keratocyte density (cell/mm²), the bright keratocyte nuclei in a predefined area (of about 0.06 mm²) were manually marked. Integrated cell analysis software was used for counting. The number of the keratocytes was derived from the average of three sections—with no motion artifact—within the 5% anterior stroma immediately beneath the epithelium.

Statistical analysis was performed using SPSS 10.0 software (SPSS, Chicago, IL). The comparisons were done with the chi-square test for categorical variables and Mann-Whitney* U* test for continuous variables. Statistical significance was considered at *P* < 0.05.

## 3. Results

The mean preoperative myopic SE refraction was −2.39 ± 1.26 D (range −1, 13 to −5.25 D) in transepithelial PRK-treated eyes and −2.52 ± 1.14 D (range −1.38 to −4.88 D) in LASEK-treated eyes (*P* > 0.05). Mean astigmatism was 1.35 ± 1.38 D (range 0.25 to 4.00 D) in 12 transepithelial PRK-treated eyes and 1.44 ±1.23 D (range 0.25 to 3.50 D) in 12 LASEK-treated eyes (*P* > 0.05).

Mean time to epithelial healing was longer after LASEK (4.00 ± 0.43 days) than that after transepithelial PRK (3.17 ± 0.6 days) and this difference was statistically significant (*P* < 0.05). On the other hand, the mean subjective pain score on day 1 was significantly higher in transepithelial PRK-treated eyes (3.75 ± 0.87) than that in LASEK-treated eyes (1.92 ± 1.83) (*P* < 0.05). After day 1, mean pain scores were similar (Table 1).

At 1 month, 58% of transepithelial PRK and 75% of LASEK-treated eyes achieved 20/20 or better UCVA (Table 2). At 6 months, 100% of eyes achieved 20/25 in both groups. Over 90% of eyes were within ±0.50 D of emmetropia at 6 months and maintained it at 12 months (Table 3). No eye lost any line of BSCVA.

**Figure 1:**
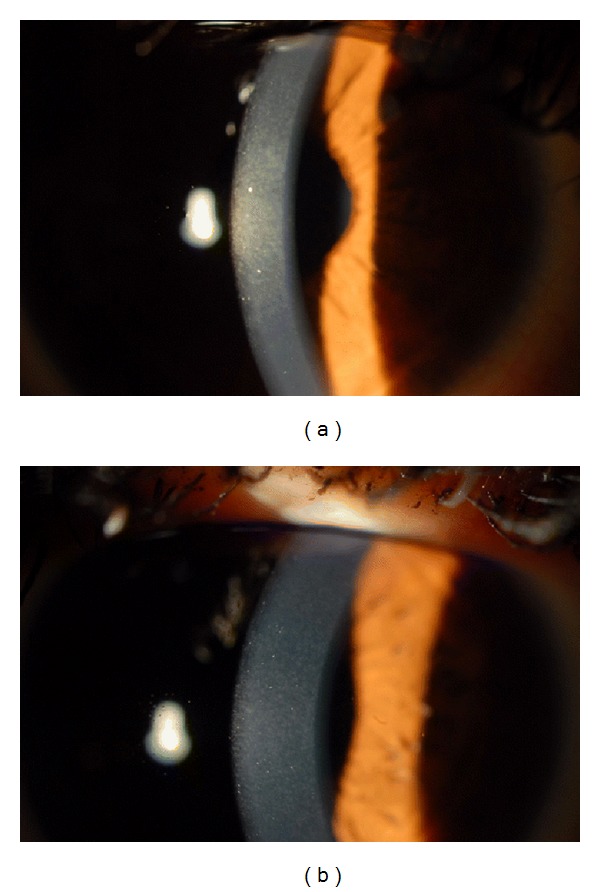
Grade 1 haze in a transepithelial PRK-treated eye (a) versus grade 0.5 haze in the contralateral LASEK-treated eye (b) of same patient at 1 month postoperatively.

**Figure 2:**
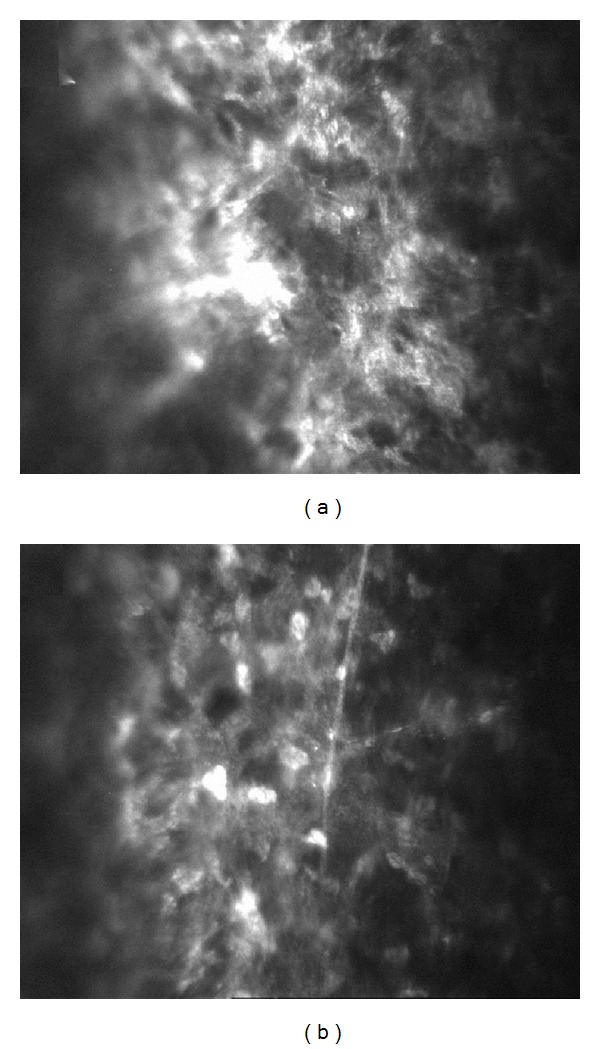
*In vivo* confocal microscopic examination of the eyes in Figure 1. In most anterior stroma beneath the epithelium, a lower keratocyte density but more extracellular matrix deposition is noted in the transepithelial PRK-treated eye (a) compared to the contralateral LASEK-treated eye (b) at 1 month postoperatively.

The mean haze grade and the mean greyscale value in confocal microscopy were significantly higher (*P* < 0.05) in transepithelial PRK-treated eyes compared to LASEK-treated eyes at 1 month postoperatively. However, the mean grade of haze and the mean greyscale value did not differ between the 2 groups after 1 month (Tables 4 and 5 and Figures 1 and 2).

The keratocyte density significantly decreased postoperatively in both transepithelial PRK and LASEK-treated eyes (Figure 2). More importantly, the keratocyte density was significantly lower (*P* < 0.05) in transepithelial PRK-treated eyes than in LASEK-treated eyes at 1 month and 3 months (Table 6). More extracellular matrix deposition and activated keratocytes were observed in transepithelial PRK-treated eyes than in LASEK-treated eyes (Figure 2).

## 4. Discussion

After a long term experience with PRK, ocular discomfort and slow visual recovery still remain the negative factors. LASEK reduced early postoperative pain compared with transepithelial PRK in our study. Lee et al. [1] believed that the reduced postoperative pain after LASEK is probably because the epithelial flap acts as another biological therapeutic lens that protects the ablated stroma from lid action. In our practice, particularly drying the LASEK flap and use of high Dk silicone hydrogel, contact lenses have provided low levels of discomfort (unpublished data). Additionally, inflammatory pain may remain limited after LASEK. In our study, complete epithelialization lagged about 1 day longer after LASEK than that after transepithelial PRK. Probably, the epithelial flap is slowly shed off and then replaced by new cells after LASEK because it loses its vitality and does not reattach completely.

Many authors found a slight difference in refractive results between traditional PRK and LASEK [1–5]. Buzzonetti et al. [8] and Luger et al. [9] reported that transepithelial PRK is safe and effective as traditional PRK for myopic correction with a minimal hyperopic shift. Lee et al. [10] reported the clinical and visual results after PRK using three epithelial removal techniques (mechanical, alcohol-assisted, and excimer laser-assisted) and found no marked difference among the three groups, as in our study. Ghadhfan et al. [11] compared the refractive outcomes and complications of LASIK, transepithelial PRK, traditional PRK, and LASEK. They detected slightly better visual results after transepithelial PRK than after LASEK and the others, but mitomycin-C application was higher in transepithelial PRK-treated eyes.

In the present study, we aimed to compare the wound healing response after LASEK with that after transepithelial PRK, which has been associated with the lowest level of wound healing response compared to other techniques of epithelial removal in PRK. At 1 month, higher haze grades and higher greyscale values in confocal microscopy were noted in transepithelial PRK-treated eyes compared to LASEK-treated eyes in our study. A greater reduction in keratocyte density was also noted after transepithelial PRK at 1 and 3 months. These results imply a more intense wound healing response after transepithelial PRK compared to LASEK.

Azar et al. [12] defined a low incidence of corneal haze after LASEK. Previous studies have also reported higher grades of haze after traditional PRK compared to LASEK, particularly in the early postoperative months [1–3, 13]. Similar to our results, Ghirlando et al. [3] reported a less intense wound healing process after LASEK than after traditional PRK, documented with confocal microscopy.

Transepithelial PRK limits initial keratocyte apoptosis and thus reduces subsequent repopulation of activated stromal keratocytes and wound healing response [6, 7, 14]. Compared with traditional PRK, transepithelial PRK have induced significantly less haze [15, 16]. On the other hand, we found significantly less haze after LASEK than after transepithelial PRK at 1 month. In contrast to our study, Lee et al. [10] did not find any significant difference among the transepithelial PRK, traditional PRK, and LASEK in terms of cornel haze. Ghadhfan et al. [11] also reported that the prevalence of haze with visual loss after LASEK and transepithelial PRK was low and comparable. But, the authors did not evaluate corneal haze within 3 months after surgery in these studies [10, 11].

A reduced wound healing process is associated with less keratocyte apoptosis, diminished production of extracellular matrix and collagen, and eventually reduced haze and regression. Confocal microscopy showed some morphologic differences in the corneal wound healing process between transepithelial PRK and LASEK in our study. Our confocal results of posttransepithelial PRK are also unique. We noted that the immediate keratocyte density was significantly lower, and then more extracellular matrix deposition and activated keratocytes were observed in transepithelial PRK-treated eyes than in LASEK-treated eyes. We believe that LASEK seems to decrease changes in stromal keratocytes and corneal wound healing up to 3 months after surgery. Animal studies have also demonstrated that LASEK provides superior results than traditional PRK in terms of keratocyte apoptosis, haze, and wound healing [17, 18]. The mechanism of how wound healing response remains less intense after LASEK is not still defined. The epithelial flap in LASEK is accepted to serve as a barrier against influx of cytokines into the stroma and impede keratocyte apoptosis, which is an essential step in wound healing. In addition to effective barrier function, epithelial flap may also minimize the reflex cytokines release originating from the lacrimal gland, regenerating epithelial and stromal cells in ablated surface following laser ablation, as suggested by Lee et al. [13].

## 5. Conclusion

Both transepithelial PRK and LASEK offer effective correction of myopia. However, LASEK seems to offer a less intense wound healing response, less haze, and less ocular discomfort than transepithelial PRK. On the other hand, time to epithelial healing is slightly longer with LASEK.

## Figures and Tables

**Table 1:** Subjective pain score after transepithelial PRK and LASEK.

Subjective pain score			
Day	Transepithelial PRK	LASEK	*P* value∗
1	3.75 ± 0.87	1.92 ± 1.83	*P* < 0.05
2	2.00 ± 1.13	1.42 ± 1.62	*P* > 0.05

*Mann-Whitney *U* test.

**Table 2:** Visual acuity after transepithelial PRK and LASEK.

Uncorrected visual acuity (percentage of eyes)					
	Transepithelial PRK		LASEK		*P* value∗
	≥20/25	≥20/20	≥20/25	≥20/20	
Preoperative^†^	100	83	100	83	*P* > 0.05
1 month	100	58	100	75	*P* > 0.05
3 months	100	83	100	83	*P* > 0.05
6 and 12 months	100	92	100	92	*P* > 0.05

^†^Best spectacle-corrected visual acuity; ∗Chi-square test.

**Table 3:** Residual refractive error (Spherical equivalent) after transepithelial PRK and LASEK.

Residual refractive error (percentage of eyes)					
Followup	Transepithelial PRK		LASEK		*P* value∗
	±0.5 D	±1.0 D	±0.5 D	±1.0 D	
1 month	83	83	92	100	*P* > 0.05
3 months	92	100	92	100	*P* > 0.05
6 and 12 months	92	100	92	100	*P* > 0.05

D: diopter.∗Chi-square test.

**Table 4:** Incidence of haze after transepithelial PRK and LASEK.

Incidence of haze (percentage of eyes)								
Followup	Transepithelial PRK				LASEK			*P* value∗
	0	+0.5	+1	+2	0	+0.5	+1	
1 month	0	42	42	16	0	92	8	*P* < 0.05
3 months	17	58	25	0	42	58	0	*P* > 0.05
6 months	50	42	8	0	67	33	0	*P* > 0.05

*Chi-square test.

**Table 5:** Greyscale value in confocal microscopic examination after transepithelial PRK and LASEK.

Mean greyscale value			
Followup	Transepithelial PRK	LASEK	*P* value∗
1 month	132 ± 64	77 ± 17	*P* < 0.05
3 months	98 ± 55	82 ± 36	*P* > 0.05
6 months	81 ± 22	70 ± 82	*P* > 0.05

*Mann-Whitney *U* test.

**Table 6:** Preoperative and postoperative keratocyte density (cell/mm²) after transepithelial PRK and LASEK.

Keratocyte density (cell/mm²)			
Followup	Transepithelial PRK	LASEK	*P* value∗
Preoperative	981 ± 66	977 ± 72	*P* > 0.05
1 month	363 ± 50	484 ± 48	*P* < 0.05
3 months	495 ± 36	586 ± 42	*P* < 0.05
6 months	601 ± 45	629 ± 52	*P* > 0.05

*Mann-Whitney *U* test.
